# Re-evaluating data quality of dog mitochondrial, Y chromosomal, and autosomal SNPs genotyped by SNP array

**DOI:** 10.13918/j.issn.2095-8137.2016.6.356

**Published:** 2016-11-18

**Authors:** Newton O. OTECKO, Min-Sheng PENG, He-Chuan YANG, Ya-Ping ZHANG, Guo-Dong WANG

**Affiliations:** ^1^ State Key Laboratory of Genetic Resources and Evolution, Yunnan Key Laboratory of Molecular Biology of Domestic Animals, Germplasm Bank of Wild Species, Kunming Institute of Zoology, Chinese Academy of Sciences, Kunming 650223, China; ^2^Kunming College of Life Science, University of Chinese Academy of Sciences, Kunming 650204, China; ^3^State Key Laboratory for Conservation and Utilization of Bio-Resources, Yunnan University, Kunming 650091, China

**Keywords:** SNP array, Dog, Mitochondrial, Y chromosomal, Autosomal

## Abstract

Quality deficiencies in single nucleotide polymorphism (SNP) analyses have important implications. We used missingness rates to investigate the quality of a recently published dataset containing 424 mitochondrial, 211 Y chromosomal, and 160 432 autosomal SNPs generated by a semicustom Illumina SNP array from 5 392 dogs and 14 grey wolves. Overall, the individual missingness rate for mitochondrial SNPs was ~43.8%, with 980 (18.1%) individuals completely missing mitochondrial SNP genotyping (missingness rate=1). In males, the genotype missingness rate was ~28.8% for Y chromosomal SNPs, with 374 males recording rates above 0.96. These 374 males also exhibited completely failed mitochondrial SNPs genotyping, indicative of a batch effect. Individual missingness rates for autosomal markers were greater than zero, but less than 0.5. Neither mitochondrial nor Y chromosomal SNPs achieved complete genotyping (locus missingness rate=0), whereas 5.9% of autosomal SNPs had a locus missingness rate=1. The high missingness rates and possible batch effect show that caution and rigorous measures are vital when genotyping and analyzing SNP array data for domestic animals. Further improvements of these arrays will be helpful to future studies.

## INTRODUCTION

Single-nucleotide polymorphism (SNP) arrays have received wide recognition for detecting DNA polymorphisms in domestic animals (Goddard & Hayes, 2009). The availability of SNP arrays to incorporate not only dense autosomal markers, but also hundreds of mitochondrial and Y chromosomal SNPs, greatly assists breeding and population history inferences (Shannon et al., 2015b). Genotyping SNPs offers superior efficiency and convenience compared with traditional Sanger sequencing or genotyping techniques, such as denaturing high-performance liquid chromatography (DHPLC) and SNPshot. Like other high-throughput techniques, however, SNP assays are not infallible. Difficulties can arise from diverse, complex, and often cryptic sources, and different factors can converge to produce an artifact (Pompanon et al., 2005). With new technological advancements in the genotyping landscape, some potential artifacts remain unknown, untested, or unaccounted for (Leek, 2014; Leek et al., 2010). Previous studies on human populations have established potential technological and experimental pitfalls in genotyping, which could compromise data quality (Palanichamy & Zhang, 2010; Peng et al., 2014). To investigate these issues in domestic animals, we performed an independent re-evaluation of recently published SNP array data representing a global dog population (Shannon et al., 2015b).

## MATERIALS AND METHODS

We retrieved dog SNP datasets from Dryad (doi: 10.5061/dryad.v9t5h) (Shannon et al., 2015a). Detailed methodology is described elsewhere (Shannon et al., 2015b). Briefly, DNA was extracted predominantly from whole blood samples by salt precipitation from 4675 pure breed, 168 mixed breed, and 549 village dogs, plus 14 grey wolves (Supplementary Table S1). The samples were genotyped against 424 mitochondrial, 211 Y chromosomal, and 160 432 autosomal SNP markers using a semicustom Illumina SNP array (Shannon et al., 2015b). We used PLINK v.1.07 to determine the missingness rates (MRs) of the datasets (Purcell et al., 2007). We analysed all individual MRs (iMR) for both mitochondrial and autosomal marker types, except for the Y chromosomal marker in females. We also calculated the locus MR (lMR) to assess the MRs for all SNPs. We used IBM SPSS statistics version 20.0 (SPSS, Inc., Chicago, IL, USA) for data analysis, and box plots were drawn by BoxPlotR software (Spitzer et al., 2014).

## RESULTS

**Figure 1:**
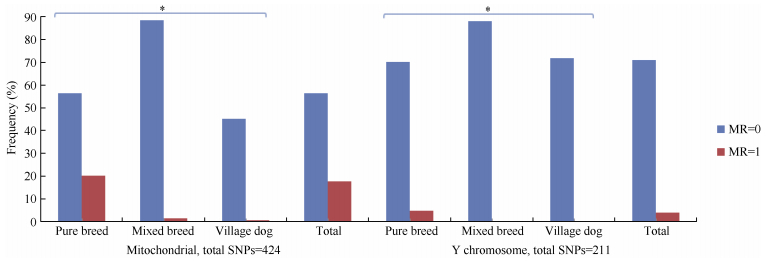
Individual missingness rates (iMR) for mitochondrial and Y chromosomal marker types

**Figure 2:**
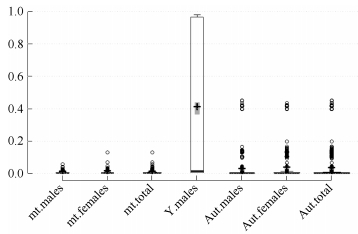
Box plot showing the individual missingness rates (iMR) for mt: mitochondrial (*n*=1 387), Y: Y chromosomal (*n*=659), and Aut: autosomal (*n*=2 744) marker types according to gender for the 0<MR<1 category

Full iMR and lMR results are shown in Supplementary Tables S2 and S3, respectively. As summarized in Figure 1, complete genotyping (MR=0) for mitochondrial and Y chromosomal SNPs was observed for 3 039 (56.2%) and 1 896 (71.2%) individuals, respectively, with 980 (18.1%) and 107 (4.0%) individuals completely missing genotyping (MR=1) for the two marker types, respectively. Pure breed dogs tended to have a higher iMR (1) than that of other dogs. Additionally, overall mean iMR values were generally higher in pure breed dogs and much higher in grey wolves, specifically for mitochondrial and Y chromosomal marker types (Table 1). This trend was mirrored in the mean iMR across breeds, excluding MR=0 values (Supplementary Table S4). All individuals recorded autosomal genotyping iMR >0 to <0.5. Combined analysis of all MR values >0 and <1 (Figure 2) showed a higher mean iMR for the Y chromosomal (>40%) than the other two markers. 

**Table 1:** Comparison of individual missingness rates (iMR) across different breed categories

	Overall iMR, mean (standard deviation)		
	Mitochondrial	Y chromosomal	Autosomal
Pure breeds	0.206 1 (0.403 4)	0.164 6 (0.365 5)	0.028 2 (0.059 9)
Mixed breeds	0.018 1 (0.132 8)	0.012 5 (0.104 9)	0.003 2 (0.014 9)
Village dogs	0.011 2 (0.085 0)	0.008 (0.053 3)	0.004 1 (0.013 6)
Grey wolves	1	0.966 8	0.130 3 (0.017 0)
ANOVA P value	0.000 1	0.000 1	0.000 1

Overall genotype missingness rates (MR>0) for mitochondrial and Y chromosomal SNPs were realized in 2 367 (43.8%) and 766 (28.8%) individuals, respectively, with the missing genotyping proportions in each breed summarized in Supplementary Table S4. Of the 980 individuals with mitochondrial MR=1, 374 were males, which all had Y chromosomal MR>0.96 (Figure 1 and Supplementary Table S5). The mean autosomal MR was also significantly higher for these 374 males (0.135) compared with the other 2 288 males (0.002) (Table 2). Further scrutiny indicated that all 980 individuals with mitochondrial MR=1 came from 1 325 samples that had a different experimental format, given the assaying plate numbering system (Sample IDs prefix, Supplementary Table S2). There was a marked difference in mean iMR across all three marker types between the two classes of samples, with those undergoing assaying plate serialization bearing lower missed genotyping rates (Supplementary Table S6). These observations suggest a likely batch effect (Leek, 2014; Leek et al., 2010) in the case of the 374 males.

**Table 2:** Comparison of individual missingness rates (iMR) for 374 males with likely batch effect versus remaining males

	Mean MR (Standard deviation)					
	Mitochondrial SNPs	*P*-value	Y chromosomal SNPs	*P*-value	Autosomal SNPs	*P*-value
In^*^	1 (0)	0.001	0.982 (0.013)	0.001	0.135 (0.069)	0.001
Out^*^	0.001 (0.003)		0.002 (0.007)		0.002 (0.003)	

^*^In=374 male individuals with likely batch effect (mitochondrial SNPs MR=1 and Y chromosomal SNPs MR>0.96), Out=other individuals (*n*=2 288).

**Figure 3:**
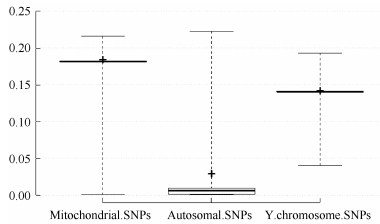
Box plot showing the locus missingness rates (lMR) for mitochondrial, Y chromosomal, and autosomal SNPs MR>0

Assessment of lMR showed that none of the mitochondrial or Y chromosomal SNPs achieved complete genotyping (MR=0). While 5.9% of the autosomal SNPs were completely genotyped, 0.5% of the autosomal SNPs together with 0.7% of the mitochondrial SNPs had a ≥20% MR among the study individuals (Table 3). Overall, lMR was higher for mitochondrial and Y chromosomal SNPs compared with that for autosomal SNPs (Figure 3).

**Table 3:** Summary of locus missingness rates (lMR) for mitochondrial, Y chromosomal, and autosomal SNPs

	Genotyping marker, No. SNPs (%)			Total
	Mitochondrial SNPs	Autosomal SNPs	Y chromosomal SNPs	
SNP MR				
MR=0	0	9 486 (5.9)	0	9 486 (5.9)
0<MR<0.1	0	134 747 (84.0)	4 (1.9)	134 751 (83.7)
0.1=<MR<0.2	421 (99.3)	15 456 (9.6)	207 (98.1)	16 084 (10.0)
0.2=<MR<0.3	3 (0.7)	743 (0.5)	0	746 (0.5)
Total	424 (100.0)	160 432 (100.0)	211 (100.0)	161 067 (100.0)

## DISCUSSION

The missingness rate can be used to clarify overall quality of genotyping. Problems at any stage of the genotyping process can adversely impact data analyses, including the definition of haplotypes and calculation of genetic diversities. Missingness rates can inform decisions on how to account for possible errors to support the genotyping process, and possibly inform technological advancements in SNP arrays (Laframboise, 2009). The observed overlapping pattern of high MR statistics for mitochondrial and Y chromosomal SNPs among the 374 males represents a possible batch effect scenario (Leek et al., 2010). 

Batch effects commonly occur in high-throughput technologies, where a subgroup of observations show qualitatively different behaviors across conditions, which might not be related to biological variability (Leek et al., 2010). Batch effects, like other genotyping problems, arise from ubiquitous sources that are often not fully recorded or reported, ranging from sample/DNA competence, date/time of experiment, technician input, reagents, chip numbers, as well as platforms or instruments used (Leek, 2014; Leek et al., 2010; Pompanon et al., 2005). Full experimental records and individual sample information, as highly advocated elsewhere (Kitchen et al., 2010; Leek et al., 2010), play vital roles in facilitating re-evaluations or meta-analyses of multiple datasets. This was a limitation encountered in our analysis, which lowered the power for definitive validation of the suspected batch effect and factors underlying high MR values.

In the present study, MRs tended to be higher for pure breed dogs than for other dogs, suggesting potential breed-based differential SNP array missingness, contrary to more robust technologies such as next-generation sequencing. Missing genotype calls are widespread in high-throughput genotyping, but their effect on subsequent analyses has been largely ignored (Fu et al., 2009; Yu, 2012). In SNP arrays, missing call rates arise from technical issues like SNP array manufacturing, DNA processing, batch size and composition, or genotype calling criteria, as well as biological issues such as previously uncharacterized variants or DNA quality and quantity (Didion et al., 2012; Fu et al., 2009; Hong et al, 2008.; Nishida et al., 2008). In addition to careful DNA quality control and quantity standardization, other mitigation measures to reduce high MRs should include employing large and uniform batch sizes in genotype calling, using homogenous samples in the same batches (Hong et al., 2008), reviewing the suitability of quality control filtering cutoffs when calling genotypes (Fu et al., 2009), and continuous characterization and inclusion of rarer genomic variants in array designs (Didion et al., 2012).

Due to the diverse, complex, and cryptic nature of genotyping issues in high-throughput technologies, such as batch effects, a thorough understanding and awareness of potential causal avenues, consequences, and mitigation strategies are serious concerns among researchers (Kitchen et al., 2010; Kupfer et al., 2012; Leek, 2014; Leek et al., 2010; Palanichamy & Zhang, 2010). SNP array technology, computational methodology, and biological inferences are closely interlinked (Laframboise, 2009). Our findings, therefore, point to the necessity of rigor and caution in the generation and use of SNP array genotyping data for domesticated animals, especially those improved for specialized traits. Continuous robustification and extensive pre-commercialization qualification of SNP arrays are areas for future consideration.

## ACKNOWLEDGEMENTS

N.O.O. thanks the support of the Chinese Academy of Sciences-The World Academy of Sciences (CAS-TWAS) President's Fellowship Program for Doctoral Candidates. G.-D.W. and M.-S.P. are grateful for support from the Youth Innovation Promotion Association, CAS.

## AVAILABILITY OF DATA AND MATERIALS

All data and software used in this paper are freely available. The SNP dataset for the 5406 dog samples has been published previously (Shannon et al., 2015b), and is freely available at: http://www.datadryad.org/resource/ doi:10.5061/dryad.v9t5h. Both the PLINK and BoxPlotR software are freely available at: http://pngu.mgh.harvard.edu/~purcell/plink and http://boxplot.tyerslab.co/, respectively. In addition, we have provided the complete missingness rate data in the online version of this article in Supplementary Table S2 (results of individual missingness rates) and Supplementary Table S3 (results of locus missingness rates) plus other supplementary results supporting this paper.
